# PRO: Testing for ESBL production is necessary for ceftriaxone-non-susceptible Enterobacterales: perfect should not be the enemy of progress

**DOI:** 10.1093/jacamr/dlab019

**Published:** 2021-05-07

**Authors:** Pranita D Tamma, Romney M Humphries

**Affiliations:** 1 Department of Pediatrics, Division of Infectious Diseases, Johns Hopkins University School of Medicine, Baltimore, MD, USA; 2 Department of Pathology, Microbiology and Immunology, Vanderbilt University Medical Center, Nashville, TN, USA

## Abstract

The MERINO trial has seemingly laid to rest the question: ‘Are carbapenems the preferred therapy for ESBL-producing infections?’ It has, however, brought another important question to the forefront: ‘How do we know when we have an ESBL-producing infection?’ A commonly used approach is the interpretation that non-susceptibility to third-generation cephalosporins (e.g. ceftriaxone MICs of ≥2 mg/L) is an accurate proxy for ESBL production. We believe that relying on antibiotic susceptibility results alone to predict ESBL production in clinical isolates is fraught with issues. Rather, we believe accurate molecular assays that detect a comprehensive range of ESBL genes, along with other relevant β-lactamase genes, are well within the reach of existing technology and necessary to optimize patient care. Herein, we elaborate on why the current approach for determining whether an organism is likely to be an ESBL producer (i) is inaccurate; (ii) encourages carbapenem overuse; (iii) ignores the potential for ESBL production in other Enterobacterales species; and (iv) promotes the silent epidemic of ESBL transmission.

Current methods available to clinical laboratories for the detection of ESBL expression in bacterial isolates are fraught with limitations. However, these limitations should not prohibit further refinement to more accurately and rapidly detect ESBLs—a task that is within easy reach of the technologies available to today’s clinical microbiology laboratories. ESBL detection is integral to the management of patients with infections caused by Enterobacterales and should not be hamstrung by technologies that were developed a quarter of a century ago.

Our colleagues suggest that third-generation cephalosporin MIC criteria (e.g. ceftriaxone MICs ≥2 mg/L) is a sufficient and suitable proxy for ESBL production among *Escherichia coli*, *Klebsiella pneumoniae*, *Klebsiella oxytoca* and *Proteus mirabilis*.^1^ We contend that this practice is misguided as it: (i) is inaccurate; (ii) encourages carbapenem overuse; (iii) ignores the potential for ESBL production in other Enterobacterales species; and (iv) promotes the silent epidemic of ESBL transmission. Below, we elaborate on each of these arguments.

## Using ceftriaxone non-susceptibility as a proxy for ESBL production is inaccurate

The use of a ceftriaxone cut-off of ≥2 mg/L to signal presumed ESBL production is sensitive, but not specific. While virtually all *E. coli*, *Klebsiella* spp. and *P. mirabilis* producing ESBLs have ceftriaxone MICs of ≥2 mg/L, not all *E. coli*, *Klebsiella* spp. and *P. mirabilis* with MICs ≥2 mg/L are ESBL producers. A portion of organisms with ceftriaxone MICs ≥2 mg/L (which remain carbapenem susceptible) will harbour plasmid-mediated *ampC* genes (p-*ampC*) or even no identifiable β-lactamase genes—and this has important treatment implications. As an example, a study including 5723 clinical *E. coli*, *Klebsiella* spp. and *P. mirabilis* isolates from 72 US hospitals in 2012 found that 87% of isolates with ceftriaxone MICs ≥2 mg/L carried *bla*_CTX-M_ genes, 7% *ampC* genes, and 6% had only narrow-spectrum β-lactamase genes.^2^ Another US study including 482 *E. coli*, *Klebsiella* spp. and *P. mirabilis* ceftriaxone-non-susceptible isolates from 2014–15 found that ESBL genes (including *bla*_CTX-M_ and ESBL variants of *bla*_SHV_ and *bla*_TEM_) were identified in 64% of isolates, p-*ampC* genes in 0.5% of isolates, both ESBL and p-*ampC* genes in 13% of isolates, and narrow-spectrum β-lactamase genes alone were identified in 22% of the 376 organisms.^3^ As a third example, amongst 293 *E. coli* and *K. pneumoniae* international ceftriaxone-non-susceptible isolates from 2014–17 that underwent WGS in the MERINO trial, 85% of isolates harboured ESBL genes, 10% possessed p-*ampC* genes and 2% had both ESBL and p-*ampC* genes.^4^ As treatment recommendations for ESBL-producing, p-*ampC*-producing and narrow-spectrum β-lactamase-producing infections differ, greater specificity than simply a ceftriaxone MIC ≥2 mg/L is needed to accurately identify and manage ESBL-producing infections.

A second concern bolstering the inaccuracy of inferring ESBL production based on ceftriaxone MICs is the uncertain supposition that ceftriaxone MIC values are reliably determined by automated susceptibility systems used in clinical laboratories. Breakpoints defined by organizations like the CLSI and FDA are based on reference broth microdilution (BMD), against which automated susceptibility test systems are calibrated. FDA criteria for clearance generally requires essential agreement (agreement of MIC) and categorical agreement of >89.9% with BMD. Data on the performance of automated systems for ceftriaxone MIC determination with contemporary isolates are not widely available; most systems were developed well before ESBL production was a concern. Over- and underestimation of accurate β-lactam MICs with automated susceptibility systems is likely common. This was demonstrated in two prominent studies—MERINO and CRACKLE-2—which highlight the under calling of piperacillin/tazobactam MIC and over calling of carbapenem MICs by the use of non-BMD-based susceptibility testing approaches.^4^^,^^5^

## Using ceftriaxone non-susceptibility as a proxy for ESBL production promotes carbapenem overuse

Reporting ceftriaxone resistance alone, in the absence of information on ESBL production, provides insufficient data to clinicians as to when to consider alternatives to carbapenem therapy for Enterobacterales infections. We explored the hypothesis that ESBL reporting might lead to more responsible carbapenem use in a prior study. ESBL production was identified (but not reported to clinicians) using an ESBL ETEST^®^ (bioMérieux) for *E. coli*, *K. pneumoniae*, *K. oxytoca* and *P. mirabilis* clinical isolates with ceftriaxone MICs ≥2 mg/L (and carbapenem susceptible) collected from 668 unique patients at The Johns Hopkins Hospital over a 6 week period in 2018.^6^ Two scenarios were compared. Scenario 1 was the presumed quantity of carbapenem use had ESBL confirmatory testing been reported to clinicians—making the simple assumption that if the ESBL test was positive, a carbapenem would be prescribed and if the test was negative, an alternative agent (e.g. cefepime, if susceptible) would be used. Scenario 2 represented the actual quantity of carbapenems prescribed for this cohort of patients. In scenario 1, 21% of isolates were ESBL producing (with any indeterminate results categorized as positive) and therefore 21% of patients would have likely received carbapenem therapy. In scenario 2, significantly more patients—62%—were prescribed carbapenems. This hypothetical investigation by no means establishes the accuracy of the ESBL Etest, but it does illustrate the potential for carbapenem overuse in the absence of confirmatory ESBL testing. Inclusion of ESBL results on laboratory reports can ensure that patients who are likely to benefit from carbapenem therapy are prescribed carbapenems and may simultaneously limit carbapenem overuse in patients expected to have favourable clinical outcomes with non-carbapenem alternatives.

## Using ceftriaxone non-susceptibility as a proxy for ESBL production ignores the potential for ESBL production in other Enterobacterales species

The species-specific prevalence of ESBL production across the Enterobacterales order is unclear. While any Gram-negative organism has the potential to harbour ESBL genes, it is generally accepted that their presence is mostly limited to *E. coli*, *K. pneumoniae*, *K. oxytoca* and *P. mirabilis*. This notion is reinforced by the limitations of current phenotypic ESBL tests, which are only validated for these four species.^7^ The lack of ESBL screening for other Enterobacterales (e.g. *Serratia* spp., *Citrobacter* spp., *Enterobacter* spp.) has led to virtually no North American ESBL prevalence estimates for these pathogens. ESBL production in other Enterobacterales may not be trivial. Investigators in Pittsburgh found that 33% of consecutive *Enterobacter cloacae* bloodstream isolates from 2003–04 produced SHV-type ESBLs.^8^ Data from 2003 from Israel indicated that 42%, 24% and 14% of *Enterobacter* spp., *Citrobacter* spp. and *Serratia* spp., respectively, produced ESBLs.^9^ A study from the same year using clinical isolates from Italian patients found that 56% of Enterobacterales other than *E. coli*, *Klebsiella* spp. or *P. mirabilis* carried ESBL genes.^10^

Understanding ESBL prevalence in these organisms is necessary to guide optimal antibiotic decision-making. For example, a study from Taiwan that evaluated 217 patients with bloodstream infections caused by *E. cloacae* between 2008 and 2012 found that 63% of isolates were ESBL producing.^11^ All 10 patients with infections caused by ESBL-producing but cefepime-susceptible isolates who received cefepime died within 30 days, whereas none of the six patients with non-ESBL-producing, cefepime-susceptible infections receiving cefepime died within 30 days. Cefepime (similar to piperacillin/tazobactam) is considered suboptimal to treat ESBL-producing infections.^1^ Without the ability to test for ESBL production in Enterobacterales beyond *E. coli*, *Klebsiella* spp. and *P. mirabilis*, clinicians may have a false sense of security that ESBL production does not contribute to ceftriaxone resistance, leading to suboptimal selection of cefepime for the management of these infections.

## Using ceftriaxone non-susceptibility as a proxy for ESBL production hinders ESBL tracking and prevention efforts

The CDC estimate that the incidence of ESBL-producing Enterobacterales (ESBL-E) infections in the USA increased by 53% from 2012 through 2017.^12^ Other regions of the world experience even higher proportions of *E. coli* producing ESBLs, sometimes exceeding half of all *E. coli* infections.^13^ Reasons for the success of ESBL-E include horizontal transfer of mobile genetic elements harbouring ESBL genes, successful bacterial clones, ingestion through animal products, excessive antibiotic use, poor sanitation and human travel and migration.^14^ As an example, almost half of all Canadian travellers with diarrhoeal illness returning from Asia or Africa became newly colonized with ESBL-E.^15^ Without diagnostics to accurately identify patients colonized with ESBL-E, efforts to prevent transmission to other vulnerable patients both in community and healthcare settings will be hampered.

The question, therefore, is not whether to perform ESBL testing, but how. While many varieties of phenotypic tests for ESBL detection have been explored, directly testing for the presence of ESBL genes (versus relying on a surrogate) will invariably be more accurate. Similarly, molecular tests have focused solely on *bla*_CTX-M_, which, while the most common ESBL gene, is not the only ESBL gene. As such, expansion of molecular tests to include additional targets—at the very least, SHV and TEM ESBL variants are well within the ability of available platforms. Molecular panels commonly used in the USA include targets such as *bla*_NDM_, which, while epidemiologically valuable, remain exceedingly rare in the USA compared with non-CTX-M ESBLs. As an example, in a US surveillance study including 701 *E. coli*, *Klebsiella* spp. and *P. mirabilis* isolates with elevated third-generation cephalosporin MICs, less than 0.5% harboured *bla*_NDM_ genes, whereas 20% harboured a SHV-type ESBL.^16^ To be clear, our point is not that *bla*_NDM_ should be removed, but that relevant ESBL and p-*ampC* targets should be added. Inclusion of ESBL targets into these systems could be of tremendous clinical value. We believe settling for ceftriaxone non-susceptibility as a proxy for ESBL production is unacceptable. Rather, we believe accurate molecular assays that detect a comprehensive range of ESBL genes, along with other relevant β-lactamase genes, are necessary to optimize patient care.

## Funding

The work was also supported in part by a National Institutes of Health award K23-AI127935 to P.D.T.

## Transparency declarations

R.M.H. has received stock and salary from Accelerate Diagnostics Inc. Both P.D.T. and R.M.H. were past or present voting members of the CLSI Subcommittee on Antimicrobial Susceptibility Testing, but neither have received any financial compensation for these efforts. Neither author reports any conflicts of interest.
